# A Blueprint for Involvement: Reflections of lived experience co-researchers and academic researchers on working collaboratively

**DOI:** 10.1186/s40900-022-00404-3

**Published:** 2022-12-05

**Authors:** The Blueprint Writing Collective, Claire Fraser, Bekah Carrington, Jodie Crooks, James Diffey, Nicola Evans, Sue Kirk, Rhiannon Lane, Rose McGowan, Georgia Naughton, Steven Pryjmachuk, Keeya Saund, Rachel Temple

**Affiliations:** Manchester, UK

**Keywords:** Patient and public involvement, Co-researcher, Co-production, Lived experience, Qualitative research, Mental health, Research training, Young people

## Abstract

Patient and public involvement in health research is important to ensure that research remains relevant to the patient groups it intends to benefit. The UK NIHR funded Blueprint study aimed to develop a ‘model’ of effective service design for children and young people with common mental health problems. To ensure Blueprint’s findings were rooted in lived experience and informed by different perspectives, six young adults with lived experience of mental health issues were recruited, trained and employed as co-researchers to work alongside academic researchers . Blueprint collaborated with a third sector partner (McPin) to recruit, employ and mentor the co-researchers and deliver a bespoke training and mentoring package to support their development. Since Blueprint’s scheduled work plan was significantly impacted by the Covid-19 pandemic, planned co-researcher activities had to be adapted to accommodate distance learning and remote fieldwork and analysis. Blueprint’s co-researchers, academic researchers and a representative of McPin collaboratively used a process of reflexivity and thematic analysis to capture Blueprint’s involvement journey. We identified numerous benefits but also challenges to involvement, some of which were exacerbated by the pandemic. Navigating and overcoming these challenges also allowed us to collectively identify key guidelines for involvement for the wider research community which focus on enabling access to involvement, supporting co-researchers and optimising involvement for the benefit of co-researchers and research teams. This paper presents an overview of the Blueprint involvement journey from co-researcher, academic researcher and McPin perspectives, sharing our learning from the recruitment, training, fieldwork and analysis phases in order to inform the knowledge base on lived experience involvement and provide guidance to other researchers who seek to emulate this approach.

## Plain English summary

The Blueprint study worked with young co-researchers with lived experience to explore services in England and Wales for children and young people with common mental health problems like depression, anxiety and self-harm. Blueprint aimed to find out what services exist, how children, young people and their families find out about and access these services, what the services actually do, and whether they are helpful and offer value for money. Blueprint worked closely with McPin, a charity that works to support young people with lived experience get involved in research. Together we developed a training and mentoring package to support the co-researcher’s development and their preparation for the role which included research interviews with service users, parents/carers and service providers and data analysis. The co-researchers, research team and McPin worked together to reflect on the successes and challenges of this approach to research and the challenges of carrying out this work during a global pandemic. We have summarised what we have learnt about how best to enable and support co-researcher involvement to provide guidance to other researchers.

## Background

Patient and public involvement (PPI) in research has been defined as research that is done ‘by’ or ‘with’ PPI contributors rather than ‘for’, ‘about’ or ‘to’ them and is now widely recognised as an essential component in improving the overall quality, integrity and relevance of research [1]. Whilst the importance of PPI is widely acknowledged, the extent to which studies report detailed accounts of the PPI process varies [2] and PPI contributors’ own reflections on their experiences of involvement are often absent.

Involvement can be considered as a continuum which ranges from consultation to collaboration to user-controlled research [3]. Previous studies have demonstrated several benefits arising from co-researcher involvement, including benefits to the study itself and benefits to the co-researchers in terms of skills acquirement, self-esteem, and empowerment [4, 5]. However, several challenges surrounding participative research have also been highlighted, including the amount of time and resources required [4–6], potential power imbalances [7] and the risk of stigma which could arise through co-researchers disclosing personal health experiences [8]. When the co-researchers are also young people there can be additional gatekeeper and governance hurdles to overcome [9].

A recent scoping review of co-researcher involvement in research [10] identified a knowledge gap concerning the ethical dilemmas of including a ‘vulnerable group’ (so defined as they were young and patients) as co-researchers. This is particularly pertinent in relation to mental health related studies, where co-researchers are recruited on the basis of their ‘lived experience’ of mental health difficulties or service use. Here there may be an unspoken assumption that they will need to share such experiences in some form during the course of the research which could expose co-researchers to potential stigma [4]. Arguably such sharing needs to be carefully considered and skilfully delivered in order to avoid over-exposure of the co-researcher and detracting attention away from participants’ accounts.

Little has been written about how this dilemma is negotiated in practice and/or the way in which ‘lived experience’ identities shape actual research practice and team dynamics when lived experience is embedded within the research team. Whilst some have explored what resources and support are needed for involvement and provided reference to guidelines for practice these have focused on ‘patient engagement’ rather than co-research and lack specificity to mental health and young people [11, 12]. Furthermore, throughout our collaborative journey on the Blueprint study we were struck by how little practical guidance there is on *how* to achieve successful collaborative involvement beyond the theoretical and aspirational guidance [1, 13, 14] and this was further illustrated by the many requests we received from other research teams who wished to emulate our approach on Blueprint but lacked guidance in how to do so, particularly in relation to collaboration with young people.

Thus, using involvement during a qualitative study exploring services for young people with common mental health problems as the context, this paper provides a reflective account of lived experience co-researchers’ and academic researchers’ expectations and experiences of involvement thus adopting an approach which incorporates ‘two-way learning as an outcome of involvement’ [15] in order to share guidelines (Box 1) to support the involvement of young co-researchers on future projects.

**Box 1:** A Blueprint for Involvement

*Enabling access to involvement*
• Wherever possible, co-researchers should be recruited at project inception and employed throughout and sufficient time and funding allocated to navigate governance procedures and to provide training and mentoring support
• Recruitment for co-researcher roles should emphasise the value that lived experience can bring to a project rather than focusing on formal qualifications
• Recruitment should aim to engage with a broad range of young people with lived experience by advertising roles beyond existing networks and recruitment channels
• Applicants for co-researcher roles should be reassured that they will not be expected to disclose details of their lived experience
• Recruitment should emphasise the provision of training for co-researchers such that previous research experience is not always necessary
• Recruitment should emphasise the potential benefits of involvement to co-researchers such as what they can gain from the role
• Co-researcher roles should incorporate flexibility in the hours worked and the methods of involvement to ensure the role is manageable to those with other commitments such as study, work or caring responsibilities in order to broaden access
• Research teams need to be flexible to fit in with co-researchers’ other commitments and offer to hold catch up meetings with co-researchers who are unable to attend a scheduled project meeting
• Employing a cohort of co-researchers can assist with providing flexibility of involvement whilst meeting the needs of the project and offering a supportive peer environment
• Governance procedures (e.g., NHS research passport approvals) should be reviewed to facilitate involvement of young co-researchers without unnecessary bureaucratic delays
*Supporting involvement*
• Training in research methods should be provided to enable co-researchers to feel confident in the role and to enable a full contribution to be made. Training should incorporate both taught and practical sessions to aid preparedness for the role
• Training should incorporate a session on how lived experience might inform the role and to provide an opportunity to discuss expectations, appropriate sharing of information and any concerns regarding lived experience status. Inviting an experienced service user researcher to share experiences is recommended
• Ongoing learning and support for professional development should be encouraged by providing access to organisational resources wherever possible
• Training may need to be tailored to meet the specific needs of a cohort or to provide additional support sessions as needed. An initial ‘getting to know each other’ session to check on previous experience and level of research knowledge can be helpful in tailoring sessions for a specific cohort as well as building cohesion in the group
• Training sessions should also facilitate the cohort to develop as a group and to build in peer support strategies, for example, buddying up in pairs. If the training is being delivered remotely the use of break out rooms are helpful in this process
• Mentoring support should be provided alongside research methods training to ensure co-researchers are fully supported and nurtured throughout their involvement. It may be helpful to partner with a specialist organisation to provide this role and this should be costed in during project planning and sufficient time allocated to complete any contractual requirements
• Mentoring and support needs should be determined on an individual basis for each co-researcher and regular one-to-ones should be provided
Regular meetings should be held between a representative of the research team and the person/organisation responsible for mentoring to facilitate updates on project progress and co-researcher tasks and to ensure the research team are aware of any additional support needs or breaks needed for co-researchers
• Delays should be avoided between research training and related project tasks. It might be helpful to stagger training throughout the project to align specific modules with different phases of the project. Where delays are unavoidable refresher sessions should be held
*Optimising involvement*
• Choice and flexibility about the level of involvement and the ways in which co-researchers can contribute to a project should be offered wherever possible. Using an opt in approach for each project task can facilitate this process
• Regular contact should be maintained between the research team and the co-researchers as well as regular mentoring support to enable a connection to be maintained even during quieter times on the project (e.g., when waiting for governance approvals prior to data collection commencing)
• It can be helpful to reach agreement as a collective team about how and when communication will take place, e.g., weekly, fortnightly, by email, by Zoom etc. and to alternate meeting days and times to accommodate co-researchers’ other commitments
• Hybrid working should be adopted where possible to meet a range of preferences in working environment and to allow the role to fit with other commitments in order to broaden access
• Hybrid approaches to co-involvement data collection are sensible, particularly when there are few co-researchers employed. For example, specifying in governance documentation that researchers may be accompanied by a co-researcher during participant interviews so that interviews can go ahead even when the co-researcher is not available in order to prioritise research participant availability
• Research teams should be clear from the outset about contracted hours and any funding limits which may impact on involvement at various stages. Wherever possible funding should allow involvement throughout the project from inception to dissemination
• Remuneration for co-researcher roles should be commensurate with the equivalent researcher role, for example, matched to a typical University Researcher scale depending on the level of experience of the candidates in order to ensure parity of status
• Co-researchers should be supported on their journey to involvement in data collection. Initially observing a more experienced researcher undertake an interview can be very helpful
• Meeting prior to co-interviews is essential to plan how the interview schedule will be divided and to ascertain any additional support needs for the co-researcher. Sessions following interviews are useful to provide a shared opportunity for debrief and reflection, to discuss any additional support needs and to plan the level of involvement for subsequent interviews
• Some co-researchers may eventually feel confident enough to lead interviews or to progress to individual interviewing and should be supported to do this where it is appropriate for the project and co-researcher and governance procedures allow
• Research teams should build in time to reflect on the process of co-involvement in order to learn from their experiences and to help co-researchers think about the impact of their involvement and how they might utilise their experiences going forward
• Future research should seek to explore the views of research participants on their experience of being interviewed by young co-researchers and whether the involvement of lived experience co-researchers influenced their decision to take part in the research

### Description of the study, research team and nature of co-researcher involvement

The Blueprint study [16] aimed to develop a ‘model’ of what services for children and young people experiencing common mental health problems should look like by exploring what makes services accessible, acceptable and effective. The Blueprint research team comprised academics (SK, NE, SP) who were all co-applicants on the NIHR awarded grant and two academic Research Associates (CF, RL) who were appointed after funding was awarded. PPI permeated Blueprint from inception through to dissemination. From the very start the research team felt strongly that the study findings would have more validity and credibility if young people with lived experience were actively involved in the research – i.e., as co-researchers involved in data collection and analysis—rather than only providing advice and guidance to the study.

To this end, the research team decided that the fieldwork (primary research) aspects of the study should be supported by ‘young co-researchers’ working alongside the study’s two substantive research associates to collect and analyse qualitative data at case study sites (services across England & Wales for children and young people with common mental health problems) and sought funding during the grant application stage to facilitate this involvement. Whilst the Blueprint project was focused on services for children and young people aged 0–18 years, it became clear early in the project that employing and gaining governance approval for young people under 18 years to be involved in data collection would be problematic. A decision was therefore made to recruit young adults (aged 18–25 years) with lived experience of children and young people’s mental health services since this avoided the governance issues associated with under 18s while the lived experiences would still be relatively recent.

The research team were clear from the outset that the co-researchers should have equal status as the members of the research team and that their remuneration should thus be in line with university research assistant pay scales in order to help reduce any power imbalances. To achieve parity in status, the co-researchers would also need research training and mentoring support. These costs were included in the grant application and we had anticipated that the co-researchers would be employed by the host universities. However, the research team faced significant bureaucratic challenges in trying to achieve this in practice due to challenges in relation to HR proposed employment status (‘casual worker’ rather than employee) which would then impact on documentation deemed necessary by the contracting NHS trust (e.g., contract of employment) for governance approvals. Challenges were also evident in relation to who might take responsibility for DBS checks and by organisational limits on the length of casual worker contracts (13 weeks). We thus began to explore a solution for employing the co-researchers outside of the host universities or contracting NHS Trust and subsequently forged a partnership with the McPin Foundation (‘McPin’), a research-focussed mental health lived experience organisation [17]. McPin were considered an ideal partner for the Blueprint study as they are a registered charity which works to support young people with lived experience to get involved in research. McPin were contracted on a consultancy basis to recruit and employ the co-researchers and to provide support and mentorship for the co-researchers throughout their involvement in the project. Mentoring support was included based on McPin’s previous work in this area with young people and to ensure that the co-researchers had an opportunity to access support independently of the academic research team if desired. Mentoring was provided on a one-to-one basis according to need and included both emotional support and career development opportunities.

Recruitment adverts for the co-researchers were disseminated via social media and via the research team and McPin’s networks. Potential applicants were asked to complete an application form and shortlisting and interviews were undertaken by a member of the research team (CF), a McPin representative (RT) and a member of the McPin Young People’s Network who was independent of the Blueprint project. Shortlisting used an established McPin scoring system which assesses candidates based on evidence of meeting the essential and desirable job description criteria.

A decision was made to recruit a cohort of up to six co-researchers due to the geographical spread (England and Wales) and length of the study (field work over 1 year) to prevent the role becoming burdensome for one or two co-researchers. The research team’s experience of working with service user researchers on other projects also suggested a need to be mindful of flexibility in availability in order to accommodate holiday, sickness or study periods. The co-researcher posts were advertised as part-time posts with flexibility in the hours worked in order to meet the ad-hoc demands of the project over a 12-month period. The hours worked equated to approximately two days per month.

Twenty-seven applications were received and shortlisted applicants were due to be interviewed in March 2020 at McPin’s offices in London. However, this had to be postponed due to the implementation of the Covid-19 national lockdown. As it became clear that travel and other restrictions were going to be in place for some time ethical approval was sought to adapt the study for remote data collection procedures and the co-researcher job description was revised to reflect this. Previous applicants were contacted to ask if they still wished to be considered for this adapted role. Twenty-two applicants went forward to shortlisting and online interviews were offered to eight; six candidates were subsequently appointed as co-researchers.

It was originally planned to deliver a two-day in-person bespoke induction and research methods training to the cohort but this had to be adapted to be delivered online over five Zoom sessions during autumn 2020. Training modules included: introduction to the Blueprint study; the role of co-researcher and guidance on using lived experience; qualitative research; research integrity and ethics; skills practice. The training, adapted from a research methods training handbook developed specifically for PPI [18], was delivered by members of the research team who had prior experience of training and working collaboratively with service user researchers.

The co-researchers were recruited to be involved in interviewing (child and young person service users, parents and carers and service providers), analysis and dissemination, and were not involved in the development of the project aims and objectives, or stages 1 and 2 of the study (a systematic review and a mapping exercise of services in England and Wales, respectively). A different group of children and young people and parents/carers did however act as PPI advisors via Blueprint’s study advisory group and via a young person’s lived experience network (Common Room). This group provided support throughout the study and also helped develop the study. The decision to train and work collaboratively with young co-researchers was commended by the chair of the NHS Research Ethics Committee who reviewed our application for the qualitative fieldwork.

In total, Blueprint’s co-researchers were involved in 22 online interviews with service users (children and young people), parents/carers and service providers, as well as advisory group and project meetings. Three of the co-researchers also co-designed and recorded a video version of the children and young people’s participant information sheet to increase accessibility and support the recruitment of children and young people into the study. The co-researchers always conducted interviews along with the substantive researchers CF and RL and were not responsible for recruitment or taking informed consent.

To fully involve the co-researchers during the data analysis phase another online training session on qualitative thematic analysis was provided, after which a number of online group analysis sessions were held in which the academic researchers and co-researchers explored in-depth a selection of transcripts from each case study site to collectively code and explore themes emerging from the data and to contextualise the data based on the co-researchers’ lived experiences.

The co-researchers are now actively involved in helping to develop research outputs and in disseminating the findings of the Blueprint study.

### Reflections on involvement

Throughout our collaborative research journey we sought to capture our experiences as co-researchers (n = 6), academic researchers (n = 5), and 3rd sector partner (n = 1) by keeping reflective diaries in order to reflect on the challenges and successes of collaboration, moving beyond the mere practicalities of research to a more meaningful cognitive practice of reflection [19].

We initially agreed collectively to do this in an informal manner, simply capturing thoughts about the process as the project progressed. The research team included reflections from the start of the project whilst the co-researchers and McPin commenced following appointment. During the final year of the project the co-researcher cohort and the two project research associates met online to discuss how best to capture (for a wider audience) our collective experiences and the co-researchers decided that they would like to each write a structured reflective piece that could then be shared (anonymously amongst the writing team) and analysed to draw out themes to inform this paper. During this meeting we discussed what aspects might readers (both potential co-researchers and research teams) be most interested in to generate ideas but agreed that the submissions should not be overly structured. It was agreed that the research team would also each submit a reflective piece for analysis and we all used the following guide for writing: ‘Please write about anything that you think would help readers to understand your experience of being a co-researcher/academic researcher/3rd sector partner on a collaborative research project and what might be helpful to consider in future co-production approaches’.

We held a further online meeting to review the data (anonymised reflective accounts) and agreed that a thematic analysis would be undertaken on the 12 submissions by a co-researcher (GN) and an academic researcher (CF) working independently. GN and CF then met to discuss the analysis and agree on emerging themes. The draft analysis was then reviewed by the wider writing team for verification.

The following thematic reflections are thus drawn from our collective experiences which have been analysed using Framework Analysis [20, 21] a matrix-based analytic method widely used in qualitative health service research. We have also summarised our reflections into guidelines for involvement in Box 1.

Three over-arching themes emerged from the analysis which captured our experiences of the collaborative journey. These were: *enabling access to involvement; supporting involvement; reflections on the co-researcher role and the experience of collaboration.*

### Enabling access to involvement

Access to involvement can be enabled by valuing lived experience to the extent that opportunities are available for direct involvement in data collection and analysis and by valuing attributes other than formal qualifications. This in turn can increase the relevance of research and provide an opportunity to gain real world research experience. Barriers can arise due to necessary but sometimes challenging governance procedures and due to the way in which opportunities are advertised and thus the ability to reach a wider pool of potential candidates.

#### Valuing lived experience

The co-researchers described being attracted to the role as it was clear from the job description and project website that Blueprint was putting lived experience at the heart of the research. This was contrasted with less inclusive experiences in other employment opportunities and described as de-stigmatising, inclusive and valuing on a personal level. The role also provided an opportunity to get involved in something that could help make mental health research more progressive and relevant and thus lead to better outcomes for other young people experiencing mental health issues:

… one essential criterion was ‘personal experience of living with a mental health problem’. I personally found this hugely refreshing and welcoming. In the past, I have always felt the need to hide my mental health difficulties, particularly regarding employment for fear of being seen as unreliable or less able to work. (Co-R)

For McPin, an organisation that had for many years supported the involvement of lived experience voices in research, this was also a novel and exciting way to expand their extensive experience in advisory involvement work with young people (e.g., facilitating advisory groups) to a more active and direct involvement role:

The Blueprint co-researcher model was a new experience for us at McPin. Whilst we had employed co-researchers before, we had not yet explored this model for young people. Traditionally we have involved young people in research by recruiting a Young People’s Advisory Group ... Although [this] can have great impact, the co-researcher model differs slightly in that the young people’s role within the research is considerably more active; they are involved in conducting and delivering the research alongside the research team. I was incredibly excited (and nervous) about experimenting with this model for the very first time! (McPin Representative)

#### Research experience

Some of the co-researchers had wanted to gain research experience previously but had found that advertised positions often required previous experience. The co-researcher role on Blueprint was thus described by the co-researchers as a rare opportunity for a young person to gain research skills and be involved in real world research as it valued lived experience above formal qualifications and thus removed barriers which might normally be present:

The opportunity to be involved in mental-health research as a young person without masses of higher-level qualifications is seemingly difficult to come across. The Blueprint Project didn’t ask for any formal qualifications, instead focussing on experiences and qualities of candidates (Co-R)

#### Accessibility of the role

Whilst we sought to advertise the co-researcher roles as widely as possible by using social media as well as our research team and McPin networks it is clear that the cohort of co-researchers were not necessarily representative of the wider population of young people with lived experience as they were all recent graduates or in their final year of undergraduate studies. This lack of representation was recognised by both the research team and the co-researcher cohort:

We appointed a range of competent, enthusiastic and capable young people. But we have the typical problem of university-associated work in that the young co-researchers are from an eloquent and educated population with very good communication and interpersonal skills. I think they are all graduates and most already have some research experience through their undergraduate or postgraduate experiences. Of course these skills are highly desirable for the co-researcher role but I wonder how we could have tapped into those from less educated backgrounds or who are less eloquent because we may have missed out on important different perspectives and there is nothing to say that this group of young people would be any less capable in the co-researcher role. (AcR)

I wonder how accessible this role is for individuals from a diverse range of backgrounds, I personally am not sure how I could have fulfilled the role if I wasn’t a student with very flexible work hours. I’m not sure if there is any was around this when you need to use these type of contracts and fit mostly with usual working hours; as previously mentioned the team were very flexible but I am still not sure how I could have made it work if I was in full time education/ work. (Co-R)

#### Governance procedures

A significant hurdle to overcome, which could have impeded access to involvement, was the need to navigate sometimes bureaucratic (but of course in the wider context, necessary) governance procedures in order for the co-researchers to be involved in data collection at NHS sites. For example, co-researchers were required to obtain NHS research passports despite not being responsible for taking consent, having only remote contact with participants and only working under supervision from an experienced academic researcher (holding all governance approvals):

We always understood that the young co-researchers would require DBS checks but we never anticipated that some organisations would want full research passport applications for ad hoc co-researchers who would always be accompanied by a core project researcher with a research passport. Some governance departments also seemed to lack understanding of what we were doing within the project and they were clearly not geared up to having young service users as co-researchers. These problems were not insurmountable and were eventually resolved; however, they required a lot of core researcher time in chasing them up and questioning ‘the rules’. (AcR)

We were also advised during initial discussions that there were likely to be significantly more challenges to employing co-researchers (for example, in relation to insurance and risk assessments) if they were under the age of 18 years:

Everyone seems to think it’s a good idea to involve young people as co-researchers in projects about young people’s (mental) health but the governance processes don’t seem to be geared towards facilitating this. Ideally, we would have liked to have worked with, say, 14-18 year olds as co-researchers but no doubt the governance would have been even more complicated and bureaucratic. We were sensible enough to work with ‘adult’ co-researchers and use an advocacy organisation to help facilitate their involvement but even those two decisions did not prevent a whole host of bureaucratic and administrative issues. Research governance departments – especially within NHS Trusts – need to think about whether a purported service user involvement ethos extends to ensuring the bureaucratic processes they put in place to support co-researchers (if any are in place) are relatively straightforward and efficient. (AcR)

### Supporting involvement

If co-researchers and research projects are to realise the full benefits of lived experience involvement then it is important that appropriate mentoring support and training are provided and that choice and flexibility are built into the roles and responsibilities.

#### Mentoring support

The research team chose to collaborate with the McPin Foundation due to their expertise in supporting lived experience to be at the heart of mental health research and their experience in working to support the involvement of young people. As a research team we were clear that the co-researchers could always come to us with any queries but we felt it was important to provide access to independent, specialist support, particularly in relation to any emotional support needs. The success of this partnership approach to involvement with McPin was acknowledged by all members of the research team:

It has been invaluable having the support and expertise of McPin – for us as well as the co-researchers. Their expertise and experience in appropriately employing and supporting young people has given us the confidence to involve co-researchers in the study. (AcR)

The co-researchers all described feeling well supported throughout their involvement in the project due to the support structures put in place with both McPin and the research team which included research support, mentoring and access to therapeutic support. These support structures were particularly welcomed in the context of working with research participants with lived experience of mental health issues:

…caution should be applied particularly if co-researchers are required to encounter the difficult experiences of others (e.g. during research interviews). In this project, we had access to therapeutic support via McPin as well as mentoring support. Engaging with the lived experiences of others could be triggering for our own wellbeing but in this project care was taken to avoid this. (Co-R)

Key factors that seemed to further underpin support were the accessibility of mentors and research team members and the regular meetings and communication on the project which helped to maintain connections during remote working:

I never felt isolated despite working from home. It was easy to get in touch with any of the members of staff involved with the project via email if we had any questions or needed help with anything at all (Co-R)

We collectively planned the support structure to meet the needs of the co-researcher cohort and the needs of the study, agreeing how often we would meet, for one-to-one mentoring and supervision and as a qualitative fieldwork group. We felt it was important to collectively design the support structure and to build in flexibility, for example arranging additional ‘catch up’ meetings where a co-researcher had been unable to attend a scheduled meeting with the rest of the group or needed some additional one-to-one support:

Regular support was really beneficial in terms of allowing me to have the space to reflect on how I was finding the work, my goals, and areas I needed additional support. Having this support also helped me feel like a valued member of the team rather than just an occasional contributor towards the project. (Co-R)

An important element of the support structure was the regular contact between McPin and the lead contact on the research team (CF) via monthly meetings. In addition to facilitating practical elements of the project (approvals for payment etc.) it enabled the research team to be aware of any other projects the co-researchers were becoming involved in and (with the co-researcher’s consent) to be made aware of any additional support needs or times when a co-researcher needed to take some time out. The process also facilitated a regular briefing for McPin on project progress and upcoming project tasks for the co-researchers.

#### Training

The research team were able to build on experience from a previous project where a bespoke research methods programme had been developed to support service user involvement in research. In addition to the core research methods components we also built in additional components to introduce the mentoring support and to develop peer support amongst the co-researcher cohort. The content of the final training session was chosen by the co-researchers in order to meet any outstanding or specific needs. Training and support continued throughout the period of involvement, for example providing one-to-one support and feedback prior to, and following joint interviews with participants.

Feedback on the training (via an anonymous online evaluation form) indicated that it had been comprehensive and resulted in the cohort feeling adequately prepared for the role, particularly following a practice interview session. The use of break out rooms had been important to develop connections amongst the cohort in the absence of in-person training. The cohort particularly welcomed a discussion, facilitated by an experienced service user researcher guest speaker, about expectations in relation to drawing on lived experience in the co-researcher role and the opportunities they had to shape their role in the project:

The training overall felt like it comprehensively covered everything we needed for the project. I felt comfortable proceeding with the co-researcher work after the training that was provided. Having the opportunity to role play interviews especially felt like a fundamental part of the training and ensuring [sic] we felt confident going into the interviews. (Co-R)

During the training if felt like we were able to shape our contributions to the project, for example we were asked to consider what we thought our role would be as co-researchers. I think this was good as it enabled us to decide how we could have a unique impact on the project and also make the most of it for our own development. (Co-R)

#### Choice and flexibility regarding involvement

Our decision to work with a cohort of co-researchers also enabled us to incorporate more flexibility and choice into the roles and task allocation on the study. We agreed collectively that tasks arising on the study would be notified to the cohort with a choice to respond on an opt in basis and where possible, a range of ways to contribute (e.g., attending a meeting, by email). As a result, co-researchers were able to choose to be more or less involved in particular tasks at different stages of the study (e.g., some wanted to spend more time on data analysis than data collection). The key aim was to offer choice in the role, both in relation to time management alongside other commitments and the individual needs of the cohort but also to emphasise the idiosyncratic voice of lived experience:

The flexible nature of the project that was advertised was another element that attracted me to the project, hence I was able to balance my work on the project easily with my university studies (Co-R)

I also massively appreciated the fact that there were several co-researchers because this demonstrated that we were not tokens in the research project design, and that each of us brought a different lived experience and that all of those were valid and heard. I think having multiple co-researchers is important because there cannot be one person who is able to represent the views of everyone with the same background/lived experience (Co-R)

A flexible approach also brought benefits for the research project. For example, we had specified in our ethics application that co-researchers would be involved in interviews *where possible* such that research interviews could go ahead even when a co-researcher was not available to take part. This ensured that the needs of the project were also balanced with the support needs of co-researchers and any sense of burden was removed:

I think it was sensible to plan for interviews to go ahead even when a co-researcher was unable to be involved as it ensured research participant availability was always prioritised. Sometimes interviews with professionals in particular could be at relatively short notice and it was helpful to be able to just go ahead to suit the professional’s diary. (AcR)

[Just] giving us the freedom to get involved with the bits we want to and feel able to and allowing us to avoid those things we are less comfortable with or able to be involved with due to clashes, mental health or other reasons [and therefore it has] not being overly demanding or making the co-researchers feel they are individually ‘responsible’ for a whole project. (Co-R)

### Reflections on the co-researcher role and the experience of collaboration

As a writing team we all reflected on our experiences of collaboration and our thoughts about what contributed to the success of the co-researcher role and any challenges that had arisen. The themes emerging focused on project workload, teamwork, the experience of co-interviewing, sharing lived experience, remote working during the pandemic and the impact of involvement.

#### Project workload

As noted earlier, the research team set out to create an involvement opportunity that was flexible and manageable for the co-researchers alongside their other commitments with opportunities to dip in and out of project work. The co-researchers reflected that this enabled a manageable workload with no pressure to attend all sessions or be involved in all tasks and that they welcomed the opportunity to choose their type of involvement:

I found the workload was very manageable and the work we needed to do outside of the sessions was very minimal, which was ideal when balancing the project work with my university studies and personal life. Moreover, I still feel like I was involved in enough work to have benefitted from the experience as a co-researcher. There was no pressure to attend sessions, if my other commitments meant I wasn’t able to attend a meeting or training sessions the lead researchers were always understanding of this, which created a nice working environment, with no unnecessary pressures or stresses. (Co-R)

We did of course also have an allocated budget to fund this work and therefore we thought it was sensible to communicate this to the co-researchers as we sought to maintain transparency throughout the collaborative journey. In practical terms, the funding allowed for each CR to work up to 73 h on the project and we outlined this when advising that they could each make a decision about areas of the project they wanted to work on. However, it later transpired that this approach, whilst done with the best intentions, had in fact resulted in uncertainty for the co-researchers and with hindsight could have been managed better:

The only slight pressure which presented itself was the aspect of having to work under a restricted hours contract. Therefore, we had to choose how to divide our time between interviews, analysis, training and any other meetings that were offered to us to attend. It sometimes seemed hard as I didn’t want to miss out on things, but I was conscious of how many hours I had left of my contract and I was keen to make the most out of the project for myself in terms of experience and learning new skills. (Co-R)

There were times that it would have been easier to not worry about how many hours I had used/had left to use in planning future involvement. (Co-R)

#### Teamwork

One of the challenges for the research team was how to ensure the co-researchers felt fully involved and a part of the project when the funding only allowed for their part-time involvement. In many respects this issue was both challenged and mitigated by the pandemic. For example, to a certain extent we were all working in a less cohesive way due to the need to meet remotely, which may have impacted on the way the research team as a whole connected. However, the additional time gained due to the absence of travel time (to meetings and data collection sites) enabled the co-researchers to be involved in the project for a longer period of time and thus to become more embedded and to engage in a broader range of activities.

Throughout the project we sought to break down traditional academic status barriers, avoiding the use of formal titles and reinforcing the message that the co-researchers had equal status and were considered to be an extension of the research team and that we, as (older) academic researchers, had much to learn from them. In reflecting on this approach, the importance of openness on the part of experienced researchers was noted:

What specific steps can researchers take to make co-researchers feel valued and heard within the team? I guess it also requires an openness on the part of researchers to different opinions and a questioning of our assumptions and methods? (AcR)

Similarly, McPin actively involved the co-researchers in other activities beyond their Blueprint role which helped to embed their roles as part of the McPin Foundation and to broaden the experience of involvement:

Before the project started I was concerned that we may feel like token members of the team, but I found the complete opposite. I felt that our contributions were truly heard and valued. This was felt in meetings and also when it came to planning our involvement [as] we were given lots of options and could share our opinions. (Co-R)

The wider support received from McPin, for example attending team meetings, also allowed me to consider my role as a co-researcher and the unique contribution I could make to the project; it gave me an understanding of PPI outside of the Blueprint project and allowed me to reflect on the work with reference to a broader context. (Co-R)

#### Experiences of co-interviewing

Prior to the start of data collection we met with the co-researchers to help them prepare for their first interviews with study participants. Based on experiences working with trainee service user researchers on another study each co-researcher was given the option of being actively involved in asking questions from the schedule straightaway, or initially adopting an observer role with the option to ask follow up questions or probes.

In reflecting on the process of co-interviewing with the project researchers all highlighted that they had really welcomed the opportunity to initially observe and learn from the experienced study researchers as it enabled them to feel supported whilst they eased themselves into the role and built confidence:

This was a hugely valuable experience as it meant I got to learn through the process by observing experienced researchers’ interview techniques. I also found this to be really supportive and encouraging, as I was able to contribute as much or as little as I felt comfortable with. (Co-R)

The project researcher that I worked with was very understanding to the fact that I was not confident in my abilities to co-interview at the beginning. Gradually I began to ease myself into it with asking a section of the interview schedule. I would now feel fully confident to host an interview on my own. (Co-R)

Whilst co-interviewing with an experienced researcher had the benefit of ‘someone to fall back on’ and removed the pressure to singlehandedly listen to participants and plan subsequent questions, it could however, also add additional pressure for some co-researchers to ‘pick up on the right things’:

I found that co-interviewing was a successful way to conduct the interviews because I found it very difficult to continue the conversation in a meaningful way whilst also scanning the interview schedule for the next topic. Having a second researcher in the call meant that one person could be talking and fully listening while the other could be taking brief notes and/or preparing follow-up questions. (Co-R)

I personally found that there were pros and cons to co-interviewing with the project researcher. For example, an advantage of having the co-researcher is knowing that there’s someone to fall back on if I got stuck and didn’t know how to continue with a certain question. However, knowing that the lead researcher was there and knowing that it is essentially their interview, and I was in control of collecting the correct data for their study, felt like a lot of pressure for me to do the interview right. Hence, I found it at times quite nerve wracking that I wasn’t going to pick up on the right things that the lead researcher would want me to pick up on. I found these nerves sometimes gave me a sort of stage fright and I ended up performing less confidently than I knew I could, due to my worries getting in the way of my interviewing and listening skills. (Co-R)

To try and manage individual differences in levels of confidence and support needs and to ensure that co-interviews were well planned the co-researcher and academic researcher always met prior to, and after the online interviews with research participants. This provided an opportunity to plan who would lead on different parts of the interview and to debrief and provide feedback after interviews:

At the beginning of interview sessions, the project researcher and I would meet up around half an hour before to discuss the interview schedule and what I was happy to ask/take on. This extra time was so important to ensure that the interview flowed nicely. At the end of interview sessions, the project researcher would check in with myself to see if I was okay and the project researcher would give me some feedback on how the interview went. This was also very beneficial in order for me to do a mini mental health check-in and highlight areas where I can improve in my interviewing technique. (Co-R)

However, it was acknowledged that the necessity to use remote data collection procedures may have impacted on the extent to which co-interviewing could be fully interactive and intuitive:

The process of co-researching can be challenging (for both researcher & co-researcher), perhaps exacerbated by the medium we are using (remote interviews), in that, chipping in, to pick up on something the respondent has said before the researcher/co-researcher moves onto the next question on the schedule can feel so much more disruptive. Perhaps the medium affords less opportunity to probe responses? In my previous experiences of co-researching (in-person) with service user researchers the process has felt more interactive and intuitive. (AcR)

Additionally, our decision to employ a cohort of co-researchers resulted in each co-researcher having less individual opportunities to take part in interviews and thus to gain a greater breadth of research interview experience.

#### Sharing lived experience

‘Personal experience of living with a mental health problem’ was listed as an essential criterion in the job description since we considered this central to the role. However, we were conscious that ‘lived experience’ can have many interpretations (e.g., a relative of a service user could be considered to have ‘lived experience) and we did not expect candidates to explicitly define their experience, nor did we select on this basis. Equally, we were mindful that it would be inappropriate to ask candidates to discuss their experiences and thus we did not explore this other than asking, during interview, how lived experience might inform their role.

The decision to recruit young people with ‘lived experience’ was considered important by both the co-researchers and academic researchers. For example, in reflecting on this element of the role, both groups highlighted the importance of co-researcher lived experience (and age) in empathising with young participants during research interviews:

The idea of using co-researchers who have this lived experience to talk to the service users as interviewees who have similar lived experienced is a great idea, as we as co-researchers are able to emphasise with the other young people on a deeper level than the lead researchers who are older and may not have such lived experience themselves. I was therefore able to use my lived experience to show compassion and use empathetic listening skills when speaking to the service user interviewees, which I believe in a lot of research interviews isn’t the case. (Co-R)

I certainly feel the presence of the co-researchers has helped to bridge the gap at times between the academic researchers and young person participants - this perhaps relates, in part, to co-researcher age as well as the co-researcher’s lived experience. There have been some lovely interactions between co-researchers and young people in relation to shared experience of services which appears to have really put participants at ease. (AcR)

During training we invited a colleague who has spoken widely about their experiences as a service user researcher to be a guest speaker and to meet (online) with the co-researcher cohort to answer any questions and provide guidance. The co-researchers appeared to really value this session, noting that it was comforting to know that they were in the company of other colleagues with lived experience but that there was no requirement to share lived experience, for example, during interviews with participants.

It was comforting knowing that the other co-researchers in the group shared similar experiences in terms of lived experience of mental health problems. There was no pressure at all to share your experience with others, but if you did want to be more open and talk about your experience it felt like a safe, non-judgemental space to do so. (Co-R)

Discussing sensitive topics such as mental health will always present its own problems and considerations, but I felt that the research team handled it very well and emphasised that we were not required to share anything about ourselves that we were not comfortable sharing. (Co-R)

In practice, the co-researchers decided on an individual basis if and when to share their lived experience during joint interviews just as some research participants choose to share more or less of their experiences during the research process:

This same principle applied to the young people whom we were interviewing, and some participants were happy to share more than others. It was reassuring that anyone could discuss mental health problems openly without any pressure or judgement if they wanted to, but equally that it was not forced. I also greatly appreciate that, as a foundation of co-research, experience of lived mental health problems is part of who we are and shapes our perspectives, but we also have more to offer beyond our experiences with mental health. (Co-R)

#### Experience of remote working during Covid-19

As a writing team we also reflected on the process of working collaboratively during the Covid-19 pandemic. As noted previously we were in the midst of study set-up and co-researcher recruitment when the first lockdown was implemented and we, like many other research teams, were thrust into adapting our data collection and governance procedures and thus, the co-researcher role.

We were initially concerned that the adapted remote working role would have an impact on the research experience for the co-researchers, given the lack of opportunities for in-person meetings and data collection. To mitigate against this we agreed to meet regularly on Zoom and McPin provided regular mentoring support online. Whilst all indicated they would have enjoyed the opportunity for fieldwork visits, the remote working experience did in fact bring some benefits for the cohort, increasing accessibility and connections amongst the cohort, and the ability to manage the role alongside other commitments:

Working remotely during Covid-19 has been an interesting but valuable experience. However, I feel I actually prefer this way of working. For me, benefits include being able to attend meetings that I otherwise might not have (due to travel times between commitments), and the ability to regularly meet as a team, despite all working in different places across the UK. (Co-R)

Initially, the project got delayed due to the Covid-19 pandemic – however I was happy to hear that although delayed, the project could still continue. In addition, as the project was now fully remote it enabled me to still take part even though I had to relocate to a different city after I graduated. (Co-R)

For some, the remote role actually reduced the potential pressure associated with an office-based role resulting in less impact on their mental health:

Furthermore, because of my mental health difficulties, working remotely took away many pressures associated with workplace/office-based working, meaning I was able to be confident that I could deliver in this role to the best of my ability. (Co-R)

However, some found online discussions more challenging despite all our efforts to be as inclusive as possible:

I think I personally find it harder to contribute to group discussions when meeting in an online environment and it is possible this affected my work during the project but I think it has been a time of adjustment for everyone and I don’t think there is anything that could have been done to make this easier. (Co-R)

The requirement to collect data via remote methods was also considered by the research team to have impacted on the progression and confidence of some co-researchers:

I do feel it would have been a much more immersive experience for the co-researchers if we had all been working in person, particular over a number of days, at case study sites. Similarly, I feel the co-researchers would have developed confidence more quickly with the more intensive approach that in-person visits to case study sites would have afforded. The disjointed nature of the remote tech interviews (e.g., taking part in maybe one a week or less than this) may have impacted on the development of confidence that comes with being involved in a number of interviews. (AcR)

Additionally, the delays (nationally) to governance approvals due to Covid-19 resulted in an unintended delay between the training delivery and the start of data collection which impacted on preparedness for the role:

Overall, I think this [Covid-19] was very well accounted for by the team and it felt like the project mostly ran smoothly with respect to our involvement. Unfortunately, there was a large delay between the training and actually starting work due to Covid and perhaps it would’ve felt more comfortable if this hadn’t been the case but the research team made sure to regularly check in with how we felt and whether we felt prepared enough to start interviews. (Co-R)

#### Impact of involvement

The co-researchers described a range of personal and professional benefits as a result of their involvement in Blueprint including increased confidence, increased research and co-production skills, a greater awareness of mental health service delivery and research in the UK, increased career opportunities and enhanced CVs and a greater awareness of how lived experience can inform mental health research and service development:

The past year working on the Blueprint project has absolutely flown by and it has helped me more than I can describe in terms of my professional development. I have learnt so much whilst working on the Blueprint Project. This was my first experience of interviewing people and conducting qualitative research. I have since been able to take on many other mental health related roles that I do not think I would have been able to do had it not have been for the Blueprint Project. It improved my confidence tenfold. In addition, it increased my awareness of mental health services in the U.K., how we can potentially improve them, and the potential barriers young people face to accessing mental health care. I also learnt how to use my lived experience of mental health in a professional way to make others feel more comfortable. (Co-R)

It was noted by one co-researcher that the opportunities they had been given to direct their own involvement, for example, individually choosing areas of the project focus on, had increased the personal impact of involvement:

I wasn’t expecting the emphasis on which areas we personally wanted to focus on to ensure we each got the most out of the project. Retrospectively this was a really great part of my involvement and is something that I will definitely think about when considering future projects -so this is something I really appreciate now about working on Blueprint even though it wasn’t what initially attracted me. (Co-R)

As a research team we are delighted that all six co-researchers have directly used their experience on the Blueprint study to move on to new roles or further study in the fields of research and healthcare. However, it is important to acknowledge that the impact of involvement may have been constrained in part by some of the decisions made due to funding and governance requirements:

I personally feel the process of working collaboratively may possibly have been constrained by the decision (at grant application stage) for the co-researcher role to be limited to assisting with interviews, rather than them being trained to, where appropriate, and where confident to so, to lead or carry out interviews independently. This was of course a decision that was in part determined by governance procedures and the anticipated additional bureaucracy / challenges if co-researchers had been conducting interviews independently and therefore responsible for taking consent etc. I suppose at times our approach on Blueprint has felt like not one thing or the other…more than PPI but not full involvement? This is in part possibly due to the fact that the co-researchers came into post after the development of interview schedules and recruitment materials was complete due to timeframes with regards securing ethical approval. It would be preferable in future for the co-researchers to be in post from the start of the project and to be involved in all aspects of study design as well as data collection and analysis. (AcR)

Despite this, we did identify a number of benefits to study data collection that arose from the involvement of the six co-researchers including bridging the gap with young participants and asking probing questions of professionals:

A few instances emerged where co-researchers were able to think of additional questions that the [academic] researcher wouldn’t have considered. In one case, this was a more probing and potentially critical question asked of a professional that the researcher would have avoided for fear of seeming critical. (AcR)

The co-researchers also made a valuable contribution to data analysis and interpretation:

The data [analysis] sessions led to some rich discussions surrounding the issues emerging in the data; the comments generated were more evaluative and interpretative and perhaps here the lived experience expertise became more prominent (not only lived experience of mental health services but experience of being a young person and recent knowledge of school environments etc.). In one session, we looked at a transcript with a predominantly positive account of a particular service; however, the co-researchers were able to look at the outlined descriptions of the service and point to potentially problematic aspects of the service procedures and processes. (AcR)

These findings have captured the Blueprint involvement journey from co-researcher, academic researcher and McPin perspectives, sharing our learning from the recruitment, training, fieldwork and analysis phases in order to inform the knowledge base on lived experience involvement and to provide guidance to other researchers who seek to emulate this approach. Working collaboratively as a writing team to reflect on the journey and to produce these guidelines has allowed us to reflect on the factors which enable access to, and support within, co-researcher roles and the steps we can all take to optimise the experience of involvement for the benefit of co-researchers and research projects.

## Conclusions

This comment article set out to share our collective two-way learning [15] experiences of co-researcher involvement and collaborative working on an NIHR funded children and young people’s mental health project. In doing so we have strived to fill a gap in the current literature about how, in practical terms, to achieve successful of involvement of young people with lived experience. We hope that by sharing our collaborative reflections on the challenges and successes and providing detailed guidelines for involvement we can inspire more research teams to work collaboratively with young people with lived experience to actively involve them more directly in the research process.

Despite working within the constraints of a global pandemic we successfully achieved the aim of working collaboratively with young people with lived experience and as a result, greatly enhanced the quality, integrity and relevance [1] of the main study findings (currently under peer review by NIHR). The importance of providing mentoring support alongside research training and working in partnership with McPin to achieve this approach has been a key factor in achieving this success and in the positive experiences of involvement of the co-researchers. Consistent with previous research [4, 5] we identified benefits to the study itself as highlighted above and to the co-researchers, for example increased confidence, skill acquisition and career opportunities.

Previous research has highlighted challenges to involvement in relation to time and resources [4–6]. In this study we successfully anticipated and thus avoided these challenges by incorporating extensive planning and budgeting from an early stage of the grant writing process. We would also like to acknowledge the generosity of our funder which ensured that involvement was adequately resourced throughout.

We were also able to anticipate and thus address some of the challenges that have arisen in previous research such as power imbalances [7] and the risk of stigma due to sharing lived experiences [8] by incorporating training, mentoring support and by adopting an informal and inclusive approach to involvement throughout the whole project team.

However, there are always lessons to be learned and we completed our reflective process by thinking about how we might do things differently on future projects. Clearly some of the constraints we faced were unavoidable due to the pandemic and in an ideal world more of our meetings and fieldwork would have been in-person. That said, it is important to note that remote working brought some benefits for the co-researchers so we would seek to adopt a hybrid approach in future. With hindsight it would have been better to reduce the gap between training and data collection and in future we might delay scheduling training dates until after governance approvals are in place. Where delays are unavoidable we would suggest holding a refresher session prior to the start of fieldwork.

In Blueprint the co-researchers were appointed after the project documentation (e.g., participant information sheets and interview schedules) had been submitted for ethical approval in order to meet with project timelines agreed with our funder. It would however, have been beneficial for both the study and the co-researcher experience if the role had commenced earlier in the project in order that they could have been involved in developing these documents (alongside other PPI contributors via our Advisory Group).

Some of the most significant barriers to involvement we faced were due to governance procedures which are rightly in place to protect research participants. Whilst the need for involvement is enshrined in policy and guidance it is notable that many of the research governance systems are not set up to easily achieve this in practice. Our findings also resonated with previous research [9] which has found additional gatekeeper and governance hurdles when the co-researchers are young people. As a research and governance community we need to do more to address these practical and pragmatic challenges of balancing involvement and participant safety.

An important finding from our reflective process was the need to consider the individual needs of each and every co-researcher and to build in choice and flexibility into co-researcher roles to allow each person to grow and develop into the role at their own pace. Rather than outlining specific parameters of the role at the start of the project, it might be better to think of co-researcher involvement as a continuum [3]. For example, on the Blueprint project some of the co-researchers would (had governance rules allowed) have been willing and confident enough to individually interview research participants after they had co-interviewed a number of times. For others, they were clear they did not, at this early stage of their research experience, feel ready to take on this challenge and welcomed the co-interview approach. Our decision to employ a cohort of six co-researchers enabled a range of involvement in different tasks to be accommodated without any impact on the project.

In reflecting on how lived experiences might have influenced the co-researcher role and involvement on the project it is evident that individual choice and flexibility were again extremely important. Co-researchers (and of course, academic researchers) had a choice about whether or not to share lived experiences and we found in practice that this absence of expectation often generated a sense of openness. There is no doubt that lived experience brought much to the main project and whilst there was no expectation for co-researchers to share their experiences with research participants, when they chose to do so, it was evident that it brought richness to the interaction.

As a research community we also need to be mindful of finding ways to create co-researcher opportunities that are accessible to all in order to ensure that research is informed by a wide range of young people with lived experience. Our co-researcher cohort were themselves keen to acknowledge that they were all from a similar educational background (all recent graduates or in their final year of undergraduate studies) and thus wondered how accessible the role would have been for non-students or other young people who needed to work full-time or had caring responsibilities. Whilst remote working provided more flexibility, this could generate additional challenges for some young people due to digital poverty. We may need to look beyond our regular networks to share opportunities for involvement to ensure we are engaging with a broader and more representative group of young people with lived experience.

Whilst we have included the views of both co-researchers and academic researchers in this article one obvious omission is the views of research participants themselves. Future research should seek to explore the views of research participants on their experience of being interviewed by young co-researchers and whether the involvement of lived experience co-researchers influenced their decision to take part in the research and/or data shared during interviews.

We hope our decision to share our co-produced guidelines for involvement will inspire more research teams to incorporate young person lived experience collaboration into their research design. Furthermore, we seek to generate debate in the wider research and governance community about how best to optimise involvement within the realms of governance and ethical procedures which are rightly in place to protect research participants.

## Data Availability

Not applicable.

## Abbreviations

AcR: Academic Researcher
Co-R: Co-researcher
HS&DR: Health Services and Delivery Research
NIHR: National Institute for Health Research
PPI: Patient and public involvement

## References

[1] Health Research Authority: Public Involvement. 2022. https://www.hra.nhs.uk/planning-and-improving-research/best-practice/public-involvement/. Accessed 8 June 2022.

[2] Jones J, Cowe M, Marks S, McAllister T, Mendoza A, Ponniah C, Wythe H, Mathie E (2021). Reporting on patient and public involvement (PPI) in research publications: using the GRIPP2 checklists with lay co-researchers. Res Involv Engagem.

[3] McLaughlin H (2006). Involving young service users as co-researchers: possibilities, benefits and costs. BR J Soc Work.

[4] Flicker S (2008). Who benefits from community-based participatory research? A case study of the positive youth project. Health Educ Behav.

[5] van Staa A, Jedeloo S, Latour JM, Trappenburg MJ (2010). Exciting but exhausting: experiences with participatory research with chronically ill adolescents. Health Expect.

[6] Pullmann MD, Ague S, Johnson T, Lane S, Beaver K, Jetton E, Rund E (2013). Defining engagement in adolescent substance abuse treatment. Am J Community Psychol.

[7] Ozer EJ, Newlan S, Douglas L, Hubbard E (2013). “Bounded” empowerment: analyzing tensions in the practice of youth-led participatory research in urban public schools. Am J Community Psychol.

[8] Lincoln AK, Borg R, Delman J (2015). Developing a community-based participatory research model to engage transition age youth using mental health service in research. Fam Community Health.

[9] Sonpal K, Walker E, Swallow V, Brady L-M, Stones S (2019). Report on involving children and young people in research.

[10] Fløtten KJØ, Guerreiro AIF, Simonelli I, Solevåg AL, Aujoulat I (2021). Adolescent and young adult patients as co-researchers: a scoping review. Health Expect.

[11] Bellows M, Burns KK, Jackson K, Surgeoner B, Gallivan J (2015). Meaningful and effective patient engagement: what matters most to stakeholders. Patient Exp J.

[12] Kirwan JR, de Wit MPT, Frank L, Haywood KL, Salek S, Brace-McDonnell S, Lyddiatt A, Barbic SP, Alonso J, Guillemin MD, Bartlett SJ (2017). Emerging guidelines for patient engagement in research. Value Health.

[13] NIHR PPI website. 2019. https://www.nihr.ac.uk/documents/ppi-patient-and-public-involvement-resources-for-applicants-to-nihr-research-programmes/23437. Accessed: 17 Nov 2022.

[14] Involve website. 2018. https://involve.org.uk/. Accessed: 17 Nov 2022.

[15] Staley K, Barron D (2019). Learning as an outcome of involvement in research: what are the implications for practice, reporting and evaluation?. Res Involv Engagem.

[16] Blueprint study website. 2022. https://sites.manchester.ac.uk/blueprint/. Accessed: 21 July 2022.

[17] The McPin Foundation. https://mcpin.org/. Accessed: 21 July 2022.

[18] Bee P, Brooks H, Callaghan P, Lovell K (2018). A research handbook for patient and public involvement researchers.

[19] Mortari L (2015). Reflectivity in research practice: an overview of different perspectives. Int J Qual Methods.

[20] Ritchie J, Spencer L, Bryman A, Burgess R (1994). Qualitative data analysis for applied policy research. Analysing qualitative data.

[21] Ritchie J, Spencer L (2003). Qualitative research practice: a guide for social science students and researchers.

